# Nanoimprinted Materials for Nanoparticle Sensing and Removal

**DOI:** 10.3390/nano15030243

**Published:** 2025-02-05

**Authors:** Lavinia Doveri, Azhar Mahmood, Piersandro Pallavicini

**Affiliations:** 1 Department of Chemistry, University of Pavia, v. Taramelli, 12, 27100 Pavia, Italy; laviniarita.doveri01@universitadipavia.it (L.D.); azhar.mahmood@iusspavia.it (A.M.); 2 IUSS School for Advanced Studies of Pavia, Piazza della Vittoria 15, 27100 Pavia, Italy

**Keywords:** nanoparticle-imprinted polymers, nanoparticle uptake, nanoparticle sensing, silver nanoparticles, gold nanoparticles, silica nanoparticles, quantum dots

## Abstract

The booming expansion of nanotechnology poses the problem of environmental pollution by nanoparticles (NPs). The available methods for sensing and removing NPs from the environment are typically lengthy and instrumentally demanding. The recent introduction of NP-imprinted polymers (NPIPs), either as films or bulk materials, is an important step toward the simple and fast sensing and removal of NPs from water and air. Similarly to the well-established molecularly imprinted polymers, in NPIPs, an organic or inorganic polymeric material is first obtained with embedded NPs. Then, the NPs are chemically or physically removed by acting as a template, i.e., leaving a polymeric matrix with cavities of the same shape and dimensions. After the first examples were published in 2014, the literature has so far reported an increasing number of NPIPs that are capable of reuptaking NPs from water (or, more rarely, air), with remarkable size and shape selectivity. By laying an NPIP layer on a reporter (typically an electrode), devices are obtained that are capable of sensing NPs. On the other hand, bulk NPIPs can reuptake massive amounts of NPs and have been used for the quantitative removal of NPs from water. This review begins with an overview of NP-imprinted hollow capsules, which can be considered the ancestors of NPIPs, both as conception and as preparative methods. Then, the literature on NPIPs is reviewed. Finally, the possible evolutions of NPIPs are highlighted from the perspective of stepping toward their real-life, field use.

## 1. Introduction

The diffusion of nanoparticles (NPs) in the environment is an increasing phenomenon, whose boundaries are hard to recognize. Although more and more NP-containing and nanotechnology-based products are available for consumers on the market, there is no updated archive of such goods. PEN, the Project for Emerging Nanotechnology, collected such information but ended its activity in 2014 [1]. At that time, the PEN archive listed more than 1800 products sold in the markets of 32 countries [2], prevalently in the commercial areas of health and fitness, home and garden, and automotive. Silver was the most common nanomaterial (24%). Also, the clinical use of NP-based medicines is increasing, although in this case there is also no systematically updated archive. Recent review papers that collected data until 2021 reported that a total of ~100 nanomedicines were being marketed, and >500 were in clinical trials [3]. A more comprehensive view of the dimensions of the efforts in the nanotechnology area may be given by the money involved: according to Global Industry Analysts, Inc., the financial size of the nanotechnology was evaluated as USD 42.2 billion in 2020, with an expected increase to USD 70.7 billion by 2026 [4], and the estimated global market for nanomaterials was USD 7.1 billion in 2020, being expected to reach USD 12.1 billion by 2026 [4,5]. Research on, preparation of, use of, and disposal of such materials, goods, medicines, and devices are all potential sources of NP leakage into the environment. In addition, the incidental formation of nanoparticles by the friction, wearing, and combustion of bulk materials (metals, plastics, wood, carbon, and paper) must also be taken into account [4]. In this context, methods for sensing and removing NPs from the environment (water, air) are obviously highly desirable. The detection and analysis methods for NPs described in the literature include hyperspectral imagery with enhanced darkfield microscopy [6], single-particle ICP-MS [7,8], the same technique coupled with hydrodynamic chromatography [9] or with ion-exchange resin separation [10], biosensing with genetically engineered proteins [11], chronoamperometry [12], fluorimetry [13] and the use of fluorescent probes [14], and electrochemical reduction on a screen-printed electrode [15]. These methods were applied to Ag [6,7,9], Au [9,11], iron oxides [11], S [12], SiO_2_ [13], TiO_2_ [7,8,14], ZnO [1,10,15], polystyrene [7], and CeO_2_ [7,11] NPs, which were analyzed in water solutions. Methods for the removal of NPs from water are based on coagulation and sedimentation [16,17,18,19,20], or on absorption by different substrates, such as activated carbon [21], fibres, and membranes [22,23,24,25], and porous resin microparticles [26], with such methods being applied to the uptake of NPs of Ag [17,18,22,25,26], Au [22,26], polymethylmethacrylate (PMMA) [24], polystyrene [23], latex [25], ZnO [21], TiO_2_ [20], Fe_2_O_3_ [17], and SiO_2_ [17], and of carbon nanotubes [19]. The capture of NPs from the gas phase has been reported thanks to fibrous filters [27] and electrostatic precipitation [28].

Molecularly imprinted polymers (MIPs) are polymeric materials that are formed around molecules that act as a template. The removal of the latter leaves cavities in the polymer that are shape- and interaction-complementary to the template molecules and are, thus, capable of selectively reuptaking them. The synthesis and use of MIPs have been well established for decades for preparing sensors by pairing them with a reporting system (e.g., electrochemical, gravimetric, and optical) [29,30,31], or for removing molecules from a solution (e.g., pollutants from water) [32,33,34]. The research on MIPs has evolved so much in terms of recognition selectivity and tailored functionalities that they can be compared to antibodies [35,36]. Moreover, MIPs can be prepared either as bulk materials (e.g., as self-standing films) or as nano-dimensioned particles. The advantage of nano-sized MIPs is that the surface–volume ratio is increased, and consequently, improved sensitivity and increased efficiency can be obtained [37]. More recently, the MIP approach has evolved by also using as templates biological entities that are larger than molecules, i.e., proteins, viruses and even cells, to form large-object-imprinted polymers (LOIPs). Analogously to the use of MIPs as sensors, a reporting system (e.g., an electrode) is coated with the LOIP, and such an assembly is used for the fast and selective sensing of the bio-entity used as the template, again exploiting the cavity–object complementarity. While the LOIP area is still far less explored than that of MIPs, a significant increase in the publications started in 2005 [38] and the literature is already sufficiently extended that a number of reviews have been published, focused on protein-imprinted polymers [39] and virus- and bacteria-imprinted polymers [40,41].

**Scheme 1:**
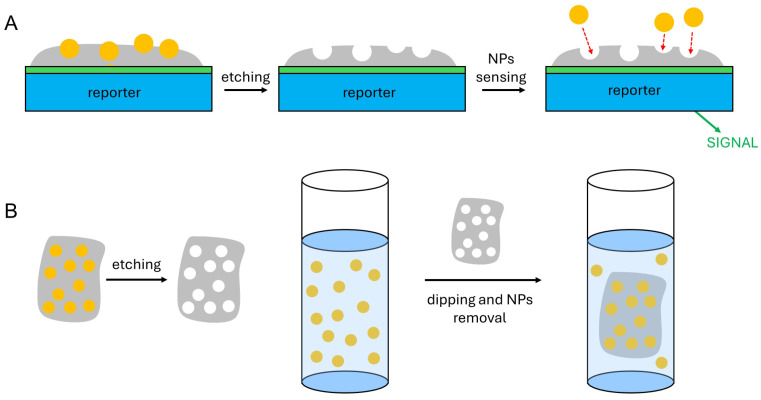
(**A**): sketch of the working scheme of a sensor built using a film of an NP-imprinted polymer; (**B**): sketch of the working scheme for the use of bulk NP-imprinted polymers for NP recovery from water.

It must be stressed that in MIPs, the template molecules have a complex shape, a number of chemical functions, and dimensions in the 0.1–1.0 range. As the polymer formed around the templates is usually obtained by crosslinking monomers that establish specific interactions (hydrogen bond, donor–acceptor, and electrostatic) with given parts or functions of the templates, the reuptake of the latter in the MIPs’ cavities is highly effective and selective [37]. On the contrary, NPs are typically highly symmetrical (spherical) objects, they have dimensions ranging from 1–2 to hundreds of nm and they are composed of a single, non-molecular substance (e.g., Au, Ag, TiO_2_, C, and CdSe). This excludes most of the shape- and function-specific interactions that make MIPs so effective in binding their substrates. However, the smaller NPs (5–50 nm) are dimensionally comparable to enzymes and proteins, and the larger ones (>200 nm) belong to the dimensional range of viruses. Also in LOIPs with protein and virus templates, the specificity of the interactions between cavities and templates is less important than in MIP. The successful studies on LOIPs indicate that the shape and dimensional complementarity between cavities and templates play a main role in the selectivity of the reuptake processes. This suggests that thin films of polymers imprinted with NPs (NPIPs) may be laid on a reporter (e.g., an electrode) to build sensors for NPs as sketched in Scheme 1A, or that bulk NPIP materials can be obtained that are capable of removing NPs from the environment, i.e., water or air, as in Scheme 1B. Despite these potentialities and an increasing number of publications, the literature in the NPIPs area is still at its dawn, as it is limited to less than 50 research papers. Most of these papers can be considered at a proof-of-concept level, i.e., demonstrating the effectiveness of the approach but not investigating important parameters such as the LOD or the detection range. In this article we will review them, stressing the challenges that NPIPs pose in the area of synthetic nanochemistry and materials chemistry. The review starts from the early studies dedicated to embedding and removing NPs in polymeric materials with the mere goal of creating empty shells of the same NP shapes and dimensions (NP-imprinted hollow capsules). The second section will be dedicated to NPIPs obtained with a matrix of organic polymers (o-NPIPs), which are typically used to obtain sensors for NPs, or with an inorganic matrix (i-NPIPs), which are used both for NP sensing and removal. The issues and potentialities of the NPIP approach in the NP sensing and recovery area are then summarized in the Section 4. For the sake of clarity, it has also to be mentioned that a vast literature has been published on what is called “nanoimprinting technology”. However, this concerns cases in which a formable or moldable material is shaped in nanoscale dimensions, e.g., using a nano-dimensioned mold prepared by nanolithography (see, e.g., ref. [42]). This is a completely different approach, and does not imply either the sensing or the removal of NPs from water, air, or whatever medium. Accordingly, this topic will not be considered in this review.

## 2. NP-Imprinted Hollow Capsules

Embedding NPs in an organic or inorganic polymeric material has been described for a large number of applications. Thanks to AgNPs’ disinfectant properties, which are due to their slow and sustained oxidation to Ag^+^, antibacterial and antimold materials have been prepared by embedding AgNPs in organic polymeric matrices, with a number of reviews available on the topic [43,44,45,46]. In the same area, photothermal antibacterial materials have been reported, obtained by embedding NIR photothermally responsive non-spherical AuNPs or Prussian Blue NPs in organic polymers [47,48]. AuNPs have been embedded also in MOFs (metal–organic frameworks) [49] or coated with silica shells [50] to obtain materials with enriched optical plasmonic behavior. Also, transparent polymers with embedded Au, Ag, or semiconductor NPs have been studied, displaying new optical properties [51]. In the sensing area, metal NPs embedded in organic polymers have been used to obtain electrochemical biosensors [52], while electrochemical and optical sensors like core–shell structures [53] or films [54] have been prepared by coating NPs with a MIP shell or by embedding NPs in a bulk MIP using semiconductor quantum dots, carbon dots, or plasmonic metal NPs.

**Figure 1:**
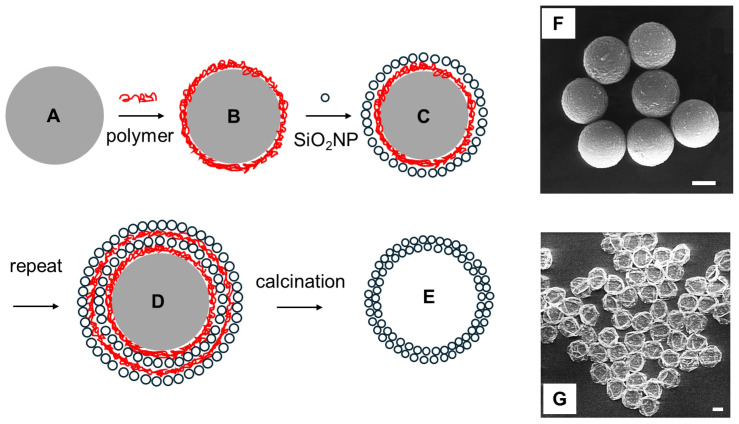
(**A**–**E**), scheme for the preparation of hollow SiO_2_ nanocapsules: (**A**) = PS nanoparticle (640 nm, negative charge), gray color in the scheme; (**B**) = same, coated with a first PDADMAC layer (positive charge), red color in the scheme; (**C**) = same, overcoated with a SiO_2_NPs layer (25 nm, negative charge); (**D**) = same, after repetitions of the PDAMMAC and SiO_2_NPs addition steps; (**E**) = SiO_2_ nanocapsule, obtained by calcination of D; (**F**) = SEM image of MF particles, coated with a PAH/PSS multilayer; (**G**) = same as (**F**), after MF template removal (images (**F**,**G**) adapted with permission from ref. [58], scale bar = 1 μm).

The process of embedding pre-prepared NPs in a plastic or rigid matrix is relatively easy, provided that the NPs agglomeration is avoided in the polymerization/solidification steps. On the contrary, the subsequent NP removal may be a critical task, especially if the target is to obtain a cavity with the same shape and dimensions as the templating NP, without damaging the polymeric matrix. In this context, smooth NP dissolution strategies that prevent disruptive reactions with the polymeric shell have been put forward to obtain hollow nanocapsules. In the pioneering work of Caruso and his group, solid NPs were used as the template. The template NPs were able to arrange on their surface a broad range of macromolecules that formed ordered architectures by self-assembly, exploiting non-covalent interactions with the NP surface. The decomposition of the core NPs followed, yielding empty nanocapsules [55]. In more detail, a diluted colloidal solution of NPs of a given surface charge was exposed to a solution of a polymer with an opposite charge until saturation. Excess polymer was separated by centrifugation and a second coating step followed using a nanometric material of opposite charge with respect to the first coating. This LbL (layer-by-layer) process could be repeated for a number of steps, allowing the control of the shell thickness. As schematized in Figure 1, polystyrene (PS) NPs (spheres, d = 640 nm, negatively charged, Figure 1A, gray) were first coated with a layer of the linear cationic polymer poly(diallyldimethylammonium chloride) (PDADMAC, Figure 1B, red color), and then with a layer of SiO_2_NPs (spheres, d = 25 nm, negatively charged, Figure 1C). The two-step process was repeated (Figure 1D) up to 5 times, reaching a multilayer thickness of 181 nm (5-time iterations). The removal of PS and PDADMAC by calcination at 500 °C yielded hollow nanocapsules (d = 720–1000 nm, for 1–5 cycles, respectively) with a wall made of tightly adhering SiO_2_NPs (Figure 1E) [56]. The nanocapsule shape was maintained after the template removal. In the same paper, the chemical dissolution of the core was also attempted using tetrahydrofuran (THF). The solvent selectively dissolved the PS template, leaving SiO_2_NPs/PDADMAC hybrid capsules. However, PS swelling during the process caused capsule fragmentation, highlighting the problematic nature of the template core removal. The LbL approach was used by Caruso and Donath also to coat weakly crosslinked melamine formaldehyde (MF) particles (d = 3.3 μM) with shells of ~20 nm thickness [57].

The positively charged MF particles were first coated with the negatively charged PSS polymer (polystyrene sulphonate), then with alternating layers of the cationic PAH (polyallylamine hydrochloride) polymer and PSS. The MF core was removed with 0.1 M HCl, leaving empty nanocapsules, with a highly flexible and permeable wall. Differently from the PS core-SiO_2_/PDADMAC nanostructures, the PAH/PSS empty nanocapsules collapsed and folded when subject to imaging in dry conditions, like in the SEM micrographs shown in Figure 1F,G [58]. Noticeably, the same approach, i.e., alternating positive/negative polyelectrolytes around an MF particle template, was used by the same group to obtain hollow capsules capable of shrinking under UV irradiation [59].

All the discussed particle templates and the obtained nanocapsules were in the hundreds of nm to micron range. The described approach was unsuccessful when polymers were used to wrap MF or PS NPs with a higher curvature, i.e., with a smaller diameter (tens of nanometers). This was mainly due to the aggregation caused by crosslinking. To overcome this problem, AuNPs with d ~ 35 nm were used as the template, the anionic thiol sodium 10-mercaptodecanesulfonate was grafted on their surface, followed by the LbL approach, alternating PDADMAC and PSS. A shell thickness of 6 ± 2 nm was obtained with 8 polyelectrolyte layers. The AuNP template core was then smoothly removed by the O_2_ oxidation of gold in the presence of the cyanide ion, according to reaction (1), which was completed in 3 h at RT [60]

4Au + 8CN^−^ + O_2_ + 2H_2_O → 4OH^−^ + 4[Au(CN)_2_]^−^ (1)

In situ polymerization has been carried out instead using a tripodal trithiol alkene grafted on 5 nm AuNPs. The removal of the gold template followed, using KCN/H_2_O_2_, to yield hollow nanocapsules [61]. Similarly, sulfur-containing polymers were grafted onto 20 nm AuNPs and crosslinked to give a sufficiently stable polymer coating that the Au core was removed with the strong oxidant aqua regia (HNO_3_:HCl 1:3 *v*/*v*) [62]. An elegant approach was used to create hollow nanocapsules consisting of a single layer of thiolated cyclodextrins. A monolayer of the latter was grafted on ultrafine spherical AuNPs (d = 2.3 nm), and then removed according to the sequence of reactions (2)–(4).

2AuSR + I_2_ → 2AuI + RS-SR (2)

I_2_ + I^−^ → I_3_^−^ (3)

AuI + I_3_^−^ → [AuI_4_]^−^ (4)

The formation of the disulfide RS-SR in reaction (2) led to inter-cyclodextrin covalent binding and to the desired hollow nanocapsules [63]. In the context of iodide used as an etching agent, ultrafine AuNPs (2.8 nm) were coated with disulfide ligands bearing an alkene function, which was subsequently polymerized. Polymer nanocapsules were then obtained by smooth AuNP dissolution with CH_3_I in DMF for 24 h at RT [64].

## 3. NP-Imprinted Polymers (NPIPs)

The production of separate hollow nanocapsules has its most interesting application in the drug delivery area, as it has been recently reviewed [65]. Continuous organic polymeric materials (thin films or bulk self-standing polymers) featuring cavities generated by the removal of embedded NP, i.e., o-NPIP, have been instead applied in the sensing area. The first examples of such materials date back to 2005. Koenig and Chechik [66] used ultrafine AuNPs (d = 2 nm) coated with a thiol ligand bearing a -(CH_2_)_11_- linear chain terminated with an acrylate function. The acrylates were copolymerized with bulk quantities of polystyrene or dimethylacrylamide and crosslinked with ethylene glycol dimethacrylate, in the presence of acetone as porogen. The removal of the AuNP templates followed, with a sequence of reactions similar to (2)–(4): using I_2_ in aqueous KI led to 2 nm diameter cavities decorated with -S-S- disulfide groups. This bulk o-NPIP was able to selectively reuptake AuNPs of comparable dimensions of the template (d = 1.4 nm AuNPs, coated with PPh_3_), while larger AuNPs (d = 13 nm, citrate-stabilized) were not able to fit into the 2 nm cavities and were not uptaken from water. Interstingly, also small AuNPs (d = 2.5 nm) coated with butanthiol were not uptaken by the imprinted materials. According to the authors, this was due to the nature of the Au-butanthiol bond, which is stronger than that between Au and PPh_3_. Accordingly, the authors proposed that the AuNP reuptake implied an interaction between the AuNP surface and the -S-S- groups decorating the interior of the cavities, which was possible only in the case of easily displaceable ligands (PPh_3_) on the AuNP surface. This first pioneering paper demonstrated the feasibility of the size-selective reuptake process in o-NPIP, but no sensing application was tried. A short communication published in 2014 introduced the use of o-NPIP in sensors, claiming the detection of 60 nm AgNPs with NP-imprinted polyurethane, polystyrene, or polyvinyl alcohol films deposited on quartz crystal microbalances [67]. A detection range (DR) between 68 and 270 ppm was reported. In the same year, the possibility of the size-selective sensing applications of o-NPIP was fully demonstrated by Mandler and coworkers in the first paper of an impressive series [68].

**Figure 2:**
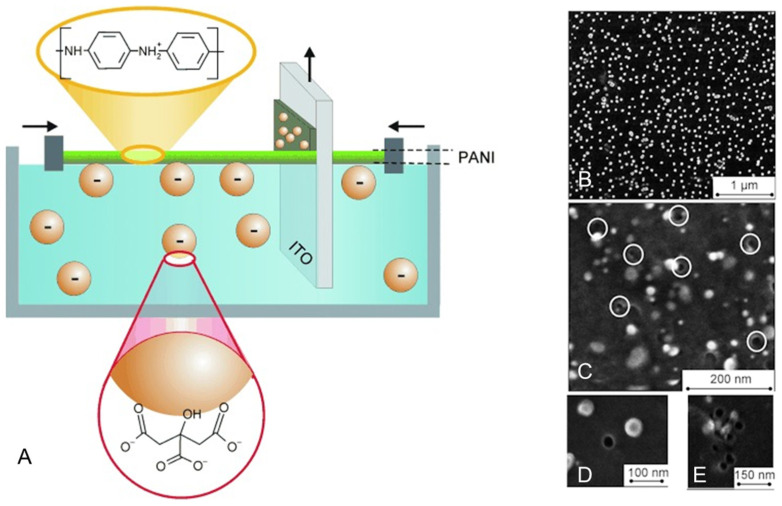
(**A**): scheme of the preparation of the ITO support coated with Langmuir–Blodgett layer(s) of PANI and AuNP; (**B**): SEM image of two LB layers with 33 nm AuNPs; (**C**–**E**): same, after the electrochemical removal of the AuNPs. Images adapted with permission from reference [68].

Negatively charged citrate-coated AuNPs with d = 15 and 33 nm were transferred using the Langmuir–Blodgett technique onto a conducting indium-tin oxide (ITO) surface, together with a positively charged monolayer of protonated polyaniline (PANI). The aqueous phase was at pH 5, so as to ensure extensive PANI protonation. ITOs bearing 1, 2, or 3 AuNPs/PANI monolayers were prepared (thickness 1–3 nm) as sketched in Figure 2A. AuNPs were then removed by electrochemical oxidation in aqueous KCl, leaving cavities of similar diameter as the template NPs (Figure 2B–E). When dipped in a solution at pH 10 (with the PANI layers unprotonated and with neutral charge), the cavities were capable of reuptaking AuNPs with size selectivity, as demonstrated by measuring the charge associated with their electro-oxidation by linear sweep voltammetry (LSV). Important observations were that (i) the size selectivity improved with the number of Langmuir–Blodgett layers (due to the attainment of deeper cavities); (ii) interactions were established also between neutral PANI (pH 10) and the negative surface of the citrate-coated AuNPs, and the authors hypothesized that the latter were hydrogen bond interactions (NH groups of PANI with COO^−^ groups of citrate), maximized by the shape and dimensional recognition in the holes; (iii) the time allowed for the AuNP reuptake before LSV was 14 h, stressing the sluggishness of the process, connected with the large dimensions of the analyte. With the same Langmuir–Blodgett technique, the same authors deposited on ITO 1 to 5 cellulose acetate (CA) monolayers with embedded AuNPs (d = 2.0 nm) coated with dodecanthiol (AuNP@C12-SH). Each CA/AuNP@C12-SH monolayer contributed to ~1 nm thickness. The AuNPs were then removed by electrochemical oxidation, leaving on ITO an o-NPIP film with the corresponding cavities. LSV showed that AuNP@C12-SH was reuptaken from a CH_3_Cl solution with increasing efficiency on increasing the number of the imprinted CA monolayers. Moreover, excellent selectivity was demonstrated in this case by maintaining the same AuNP core dimensions and changing the length of the coating thiol: heptanthiol- and octadecanthiol-coated AuNPs were reuptaken with much lower efficiency with respect to AuNP@C12-SH [69].

For the sake of clarity, Table 1 summarizes this and the following reviewed papers listing the type and dimensions of the sensed NPs, the sensing technique, the NPIP material, the property on which the sensor selectivity is based, and the range of detection.

A different approach was later introduced by the same group when an ITO surface was first functionalized with a self-assembled monolayer of APTES ((3-aminopropyl)triethoxysilane). APTES was used as an adhesive layer at pH 5 (protonated amino groups), capable of electrostatically binding citrate-coated AuNPs (40 nm diameter, negative zeta potential). On this hybrid surface, a polyphenol film of 16–19 nm thickness was deposited by electropolymerization. The removal of the embedded AuNPs was followed by electro-oxidation or by reaction (1), preserving the film and the cavity morphology in both cases. However, polyphenol is an insulating polymer, and only 18–20% of the obtained cavities were deep enough to allow contact with the electrode, as verified by cyclic voltammetry (CV) with a molecular electroactive probe. The o-NPIP surface demonstrated size selectivity in the competitive reuptake of a 1:1 aqueous colloidal solution of citrate-coated AgNPs (d = 20 nm) and AuNPs (d = 50 nm), with a significant preference for the former, which were sufficiently small to be accommodated into the 40 nm imprinted cavities (the time allowed for the reuptake was 24 h) [70]. To summarize, this working scheme, introduced by Mandler and coworkers in that paper, was as follows: (i) use a positively charged polyamine self-assembled monolayer on ITO to bind negatively charged citrate-coated AuNPs; (ii) add a polymeric or molecular filler; (iii) chemically or electrochemically remove the AuNP templates; (iv) use the obtained material to reuptake and electrochemically sense AuNPs or AgNPs by LSV. Selectivity was observed based on NP dimensions. More examples followed with a similar scheme, including the use of a monolayer of polyethyleneimine, AuNPs of 10 or 40 nm diameter, and either oleic acid or polyacrylic acid as the filler [71]; poly(diallyldimethylammonium chloride), 8 nm AuNPs, and sol−gel matrices made of various precursors [72]; poly(diallyldimethylammonium chloride), 8 nm AuNPs, and aryldiazonium salts [73]; and poly(diallyldimethylammonium chloride), 15 nm AuNPs, and 4-carboxyphenyldiazonium. In this latter case, although the template AuNPs had the same core, they featured different capping agents, namely citrate, 3-mercaptopropionic acid, and 4-mercaptobenzoic acid. It has been shown that the AuNPs with the same coating used in the imprinting process were selectively reuptaken due to complementary hydrogen bond interaction with the cavities in the o-NPIP [74]. The recognition and sensing of non-spherical AuNPs were also demonstrated along the same lines. The citrate-coated Au nanorods (AuNRs) of 47.6 ± 4.1 nm (L, length) and 12.2 ± 0.6 nm (W, width), or 34.4 ± 4 nm (L) and 12.9 ± 1 nm (W), were adsorbed on an ITO electrode bearing a positively charged polyethyleneimine layer. A non-conductive polymer was formed on ITO around the AuNRs by the electropolymerization of either 4-carboxybenzenediazonium (CBD, ionizable and negatively charged at pH > 6) or 4-acetylbenzenediazonium (ABD, non-ionizable, neutral). The AuNRs were removed by electro-oxidation, leaving rod-like cavities capable of selectively detecting AuNRs by LSV. Selectivity was observed based both on the shape complementarity between the imprinting and reuptaken AuNRs and on the polymer charge: neutral ABD-imprinted surfaces better recognized the negatively charged AuNRs with respect to the CBD negative imprinted surfaces. Moreover, it is interesting to note that shorter reuptake times, i.e., 1–4 h, were used in this research and intense LSV signals were, nonetheless, obtained. In particular, an almost linear dependence vs. time was found for the % of reuptaken AuNRs, measured as the observed LSV current vs. the LSV current of the non-oxidized matrix: 20, 30, 36, and 52% AuNR reuptake was found after 1, 2, 3, and 4 h, respectively [76].

Template NPs different from AuNPs appeared more recently in the literature for o-NPIP preparation. Schwob and coworkers used quantum dots (QDs), exploiting their intense and stable photoluminescence to study the imprinted materials and their reuptake ability. CdTeSe/ZnS QDs with elongated shapes (L ~ 10 nm; W ~ 5 nm) and coated with carboxylic acid functions were embedded in a polymer matrix by the photopolymerization of a mixture composed of methacrylic acid as the functional monomer, ethylene glycol dimethacrylate as the crosslinker and AIBN as the photoinitiator. QD removal was followed by deepening the obtained polymer (spread on PMMA plates) in aqueous acetic acid, which broke the interactions between the templating QDs and the imprinted cavities. The reuptake of the same QDs was observed from water, with dimensional selectivity with respect to larger QDs (18 × 7 nm) and with respect to QDs with the same dimensions but with a -NH_2_ terminated coating [81].

**Figure 3:**
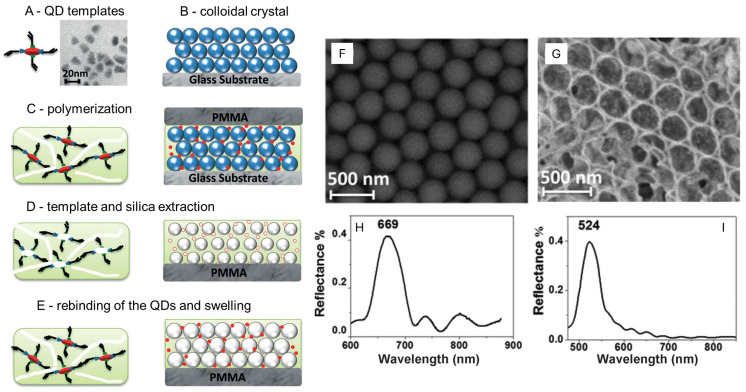
(**A**): sketch and TEM image of the QD templates; (**B**): sketch of the SiO_2_ NP colloidal crystal; (**C**): sketch of the polymerization step, with embedded QDs (red circles); (**D**): etching step (removal of both QDs and SiO_2_NPs); (**E**): rebinding of the QDs in the cavities of the PMAA polymer; (**F**): SEM image of the colloidal crystal of SiO_2_NPs, with (**H**) its reflectance spectrum; (**G**): same, of the inverse-opal structure obtained after SiO_2_ and QD etching; (**I**): reflectance spectrum. Adapted with permission from ref. [77].

The same group later developed this proof-of-concept work into a proper sensing system by following a more complex strategy, which is sketched in Figure 3A–E. An ordered colloidal crystal made of SiO_2_ NPs (d ~ 250 nm) was formed on a glass substrate using a convection method (Figure 3B) [82]. Crosslinked poly(methacrylic acid) (PMAA) with embedded carboxy-functionalized CdTeSe/ZnS QDs (18 × 7 nm, TEM image in Figure 3A) was used to fill the space among the large SiO_2_NPs, Figure 3C. Etching with HF removed both QDs and SiO_2_NPs, leaving an inverse-opal structure (formed by the SiO_2_NP voids) intercalated by a PMAA structure with imprinted small cavities of the same shape and dimensions of the QDs, Figure 3D. The rebinding of QDs took place in the complementary cavities of the PMAA polymer, Figure 3E, causing its partial swelling. Reflectance spectra were obtained from the SiO_2_NPs system (SEM in Figure 3F and reflectance spectrum in Figure 3H), but also from the inverse-opal structure of the SiO_2_NP voids, which had a highly ordered periodicity. In the latter, the position of the Bragg peak varied on the reuptake of QDs in the imprinted PMAA matrix, and a sharp correlation was found between the peak wavelength and the concentration of the QDs in a solution put in contact with such a sensing system. Size selectivity and capping agent selectivity were also observed in this case in the reuptake of QDs from aqueous solutions at pH 7. Interestingly, the time necessary for the sensing system to obtain a stable signal (i.e., the full reuptake of QDs) was 30 min [77]. LOD was as low as 0.025 ppb, with an explored detection range of 0.025–25 ppb.

A very similar approach was used to obtain a PMAA matrix containing both large SiO_2_NPs (200–400 nm) and small CdS_x_Se_1−x_/ZnS QDs (5–10 nm). The removal of SiO_2_NPs and QDs by HF etching gave a highly porous o-NPIP (the porosity was given by the SiO_2_NP template removal) decorated by small cavities (from QD removal). Such an imprinted polymer was dispersed on quartz microbalance chips, and the system was able to sense QDs in water [LOD ~ 200 ppb, range of linearity 25–2000 ppb], with selectivity based on their dimensions [78].

Also, SiO_2_ NPs were recently used as the template to obtain o-NPIP [75]. A glassy carbon electrode was activated in 0.1 M NaHCO_3_, generating -OH, =O, and -COOH functions on its surface, capable of electrostatic and hydrogen bond interactions with the NH_2_ group of amine-modified SiO_2_NPs (d = 58 ± 3 nm). Once adhering to the surface of the carbon glassy electrode, the SiO_2_NPs were embedded in a plumbagin (i.e., 5-hydroxy-2-methyl-1,4-naphthoquinone) matrix that was electrodeposited on the surface and further consolidated by the addition of a conjugate thiol (either cysteamine or 1,2-ethanedithiol), followed by the electropolymerization of the phenol ring of plumbagin. SiO_2_NPs were chemically removed by diluted HF etching, generating an o-NPIP on the glassy carbon electrode surface. Using the LSV technique, the o-NPIP-coated electrode worked as an electrochemical sensor for citrate-coated AuNPs with very similar dimensions of the cavities (d = 55 ± 1 nm; time allowed for the reuptake: 5 h). As polyphenol is an insulator, only the AuNPs fitting in the cavities were oxidized to Au^3+^ (and thus sensed) in the LSV experiments. Interestingly, this paper stressed the role of the chemical functions added to the polymer matrix in the AuNPs uptake. These were cysteamine, 1,2-ethandithiol, or nothing, decorating the imprinted polymer cavities with -NH_2_, -SH, or no functional group, respectively. At pH 5, the highest uptake of AuNPs was found for the cysteamine-modified o-NPIP, thanks to the electrostatic attraction between the protonated amino groups and the citrate anions coating the AuNPs.

Recent developments in the electrochemical detection of NPs with electrodes modified with o-NPIP included the sensing of non-conductive and non-oxidizable NPs. In a paper by Mandler’s group, SiO_2_NPs (d = 30 nm) were detected on a gold electrode coated by o-NPIP, using [Fe(CN)_6_]^3−^ as a reporting redox molecule. The o-NPIP coating was obtained in four steps: (i) the absorption of cysteamine on the gold electrode surface by Au-S bonds; (ii) adhesion on the cysteamine-coated electrode of the negatively charged SiO_2_NPs, promoted by the electrostatic interaction with the protonated amino groups of cysteamine; (iii) SiO_2_NPs embedding in a polymeric matrix of phenol or 3-aminophenol; (iv) SiO_2_NP removal by HF etching. The cavities formed in the o-NPIP allowed the contact of the [Fe(CN)_6_]^3−^ electroactive species in solution with the Au electrode. The current intensity decrease in [Fe(CN)_6_]^3−^ in square wave voltammetry was correlated with the reuptake of insulating SiO_2_NPs from aqueous solution, closing the cavities and preventing the contact of [Fe(CN)_6_]^3−^ with the underlying gold electrode. Noticeably, the best results were observed with the 3-aminophenol imprinted matrix, most probably thanks to the increased electrostatic affinity of the SiO_2_NPs to be reuptaken with the amino groups in the matrix, which were protonated at the working pH. Very importantly, from the sensing point of view, this was a faster sensor than all those presented before, as the SiO_2_NP reuptake time necessary to observe a significant [Fe(CN)_6_]^3−^ signal variation in square wave voltammetry was in the order of 2–4 min [79].

**Figure 4:**
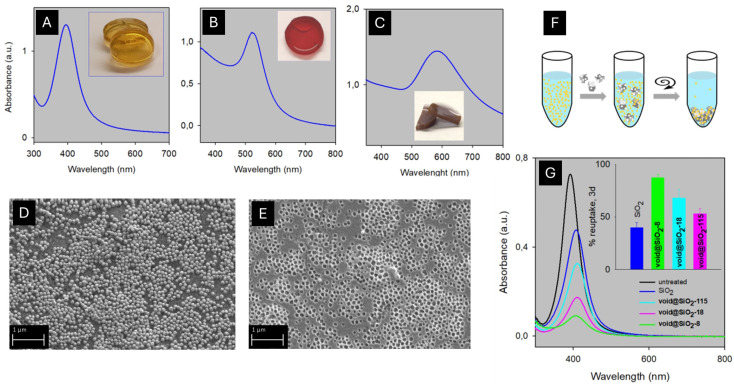
(**A**–**C**): absorption spectra of the SiO_2_ monoliths with embedded 8 nm AgNPs, 18 nm AuNPs, and 115 nm AuNPs, respectively (inset shows photographs of the monoliths); (**D**): SEM image of SiO_2_ with embedded 115 nm AuNPs; (**E**): same, after NP oxidation with aqua regia; (**F**): scheme for 8 nm AgNP removal; (**G**): absorption spectra of the supernatant of an 8 nm AgNP colloidal solution after 72 h contact with imprinted SiO_2_ and centrifugation: black, reference (untreated solution); blue: treatment with plain silica; azure: silica with 18 nm cavities; magenta: 115 nm cavities; green: 8 nm cavities. The inset displays the % of reuptaken 8 nm AgNPs. Adapted from ref. [87].

The use of inorganic polymers offers an alternative to the o-NPIP, with possible advantages particularly regarding the reduced reactivity of the polymer matrix with the reactants used to remove the template NPs, or its stability in the case of the physical removal of the latter (e.g., calcination at high temperature). Moreover, rigidity could be advantageously higher with respect to soft “plastic” organic materials, both considering self-standing polymers and thin films cast on surfaces. This aspect may be relevant in view of keeping control of the shape and dimensions of the cavities created after the removal of the templating NPs. SiO_2_ is a suitable material for all these aspects, as it is inert to most chemicals (except HF) and it is stable even if exposed to high temperatures. As a matter of fact, the only three examples of i-NPIP reported so far in the literature use silica as the matrix to obtain nanoimprinted inorganic materials. In the framework of research aimed at creating molecularly imprinted bulk SiO_2_ materials with controlled internal functionalization of their cavities [83,84,85], Katz and coworkers prepared the first silica i-NPIP. Following a procedure first described by Liz-Marzan [50], they synthesized core–shell Au@SiO_2_NPs (Au core ~ 12.5 nm) using (3-aminopropyl)trimethoxysilane (APTMS) as the coater and initiator of the silica gel formation around the AuNPs (NaSiO_3_ was the silicate source). Then, the Au/SiO_2_ core–shell NPs were embedded into bulk silica monoliths. By crushing this bulk material into 40–200 μM size particles and removing Au by O_2_/CN^−^ in aqueous solutions as described in Equation (1), they obtained SiO_2_ i-NPIP. Interestingly, the O_2_/CN^−^ Au oxidation took place also inside large SiO_2_ fragments (>200 μm), demonstrating that the intrinsically porous structure of silica gel allows the diffusion of reactants in the bulk of this material, although this may be a slow process (AuNP oxidation was complete in more than 6 h). After this, the authors studied the -NH_2_ functions inside the imprinted cavities, finding a lower number with respect to the used AuNP/APTMS stoichiometry, but they did not investigate the ability of this material to reuptake NPs [86]. On the contrary, and using a different synthetic approach to obtain SiO_2_-based i-NPIP, a very recent paper by Pallavicini and coworkers focused on the recovery of ultrafine AgNPs (d = 8 nm) from water [87]. Ultrafine AgNPs have d = 5–10 nm, and are frequently used to obtain antimicrobial materials thanks to their easy synthesis [88] and high microbicidal efficiency [89,90,91], thus raising the issue of their leakage in waste waters. Spherical AgNPs (d = 8 nm) and AuNPs (d = 18 and 115 nm) were prepared in water, capped with HS-PEG (mw 750 for AgNPs, mw 2000 for AuNPs)), and concentrated up to 1.8 mg Ag/mL and 4.7 mg Au/mL, respectively. These highly concentrated NP colloidal solutions were used to prepare SiO_2_ bulk monoliths by the addition of TMOS (tetramethyl orthosilicate) in basic water/ethanol mixtures. Absorption spectra carried out on the silica monoliths did not show a significant shift or widening of the LSPR absorptions band with respect to the NP colloidal solutions, indicating the excellent separation of the NPs in the solid matrix, Figure 4A–C.

The removal of the AgNPs proceeded on the monoliths with different straightforward etching strategies, including very soft ones like O_2_ in the presence of 0.1 M cysteamine hydrochloride [92] or 0.1 M Fe^3+^, see Equations (5) and (6)

2Ag + ½O_2_ + 4HS(CH_2_)_2_NH_3_^+^ ⇌ 2[Ag(S(CH_2_)_2_NH_3_)_2_]^+^ + 2H^+^ + 2H_2_O (5)

Ag + Fe^3+^ ⇌ Ag^+^ + Fe^2+^ (6)

The removal of AuNPs was instead carried out using aqua regia. Although the latter is a highly aggressive method, the silica matrix and the cavities structure were not damaged, as it is shown in Figure 4D,E. The reuptake ability was quantitatively determined using crushed fragments of the i-NPIP suspended in a colloidal solution of citrate-coated 8 nm AgNPs, centrifugating the large imprinted SiO_2_ fragments and their AgNPs cargo, and measuring the residual LSPR absorption in the supernatant, a procedure sketched in Figure 4F. The materials showed size selectivity, as it is shown in Figure 4G: after 72 h of contact with 8 nm AgNPs, silica imprinted with 8 nm cavities was able to reuptake 87(3)% of them, while with silica imprinted with 18 and 115 nm cavities, the reuptake of 8 nm AgNPs was 68(7) and 53(4)%, respectively. Interestingly, non-imprinted SiO_2_ was also able to reuptake 8 nm AgNPs, although much less efficiently (40(4)%). The reuptake experiments were carried out at pH 5–6, a value at which both SiO_2_ and citrate-coated AgNPs have a negative zeta potential, indicating that electrostatic interactions do not have a predominant role between SiO_2_ and AgNPs, and reuptake is dominated by Van der Waals attractive forces, which are maximized when the cavities and the NPs have the same dimensions. In this regard, a recent computational paper showed that the shape complementarity between NPs and cavities may also lead to depletion attraction, which has an entropy origin and does not require the existence of specific (coordinative, electrostatic, and H-bond) interactions between NPs and cavities for the uptake process to be effective [93]. Finally, the paper of Pallavicini and coworkers [87] evidenced also the intrinsic slowness of the landing process of NPs into cavities. While a significant (~20%) uptake of 8 nm AgNPs was observed after 1 h of contact with SiO_2_ with the cavities of the same dimensions, the reuptake process was complete only after 72 h.

All the articles reviewed so far in Section 2 and Section 3 were focused on the detection or removal of NPs dispersed in water. While water is the most probable medium in which NPs could end up in the environment, also fast, the direct instrumental detection of NPs in the air is an appealing task. In this regard, an example of a sensor for air-dispersed SiO_2_NPs recently appeared in the literature. The experimental strategy was based on the change in the capacitance of an interdigitated gold electrode. Latex NPs (d = 100 nm) were spin-coated in the space between the electrode Au pattern and then embedded in a SiO_2_ sol–gel matrix. The removal of the latex NPs by calcination (250 °C, 1 h) left an imprinted SiO_2_ i-NPIP. The docking of NPs within the cavities modulated the i-NPIP dielectric constant and the device capacitance. The electrode was exposed to aerosolized SiO_2_NP of 30, 50, 100, and 200 nm diameter, observing a capacitance increase that reached a plateau after 15 min. Noticeably, the device was selective towards 100 nm SiO_2_NP, i.e., having the same dimensions as the imprinted cavities [80].

## 4. Conclusions and Perspectives

The field of NP-imprinted polymers (NPIPs) is an emerging research area, in which the first papers describing this kind of materials were published in 2005, followed by the first applications in sensor science almost ten years later. Since 2014, an increasing number of articles appeared in the literature. However, relatively few papers (less than 50) have been published so far (end of 2024) [94]. The research background in the area of NP-imprinted hollow capsules [55,56,57,58,59,60,61,62,63,64,65] has helped to suggest techniques for building thin films or self-standing polymers with embedded NPs and for removing them, obtaining cavities with the same shape and dimensions as the template NPs. By using thin films (frequently in the range of 1–5 monolayers) [67,68,69,70,71,72,73,74,76] of o-NPIP layered on or between electrodes, it has been thoroughly demonstrated that these can work as sensors that are selective for NPs of similar shape and dimensions of those used as the template. While in the first published examples of these devices, the time needed to reach device saturation (i.e., a constant reporting signal) seemed too long to be an issue (6–24 h range), the more recent results point towards electrochemical and optical sensors for NPs that answer in a few minutes [77,79]. In the future, these can become devices suitable to monitor the NP presence and concentration both in water and in the air.

Sensors have been described for the most common types of NPs, i.e., AuNPs, AgNPs, SiO_2_NPs, and QDs. However, except for the example of silica imprinted with latex NPs and then used for sensing SiO_2_NPs [80], the literature described studies in which the imprinting and sensing NPs were made of the same material (or of very similar ones, like Ag/Au). One of the important future perspectives for o-NPIP- and i-NPIP-based devices should be to demonstrate that a given sensor can detect NPs of different nature, provided that there is a shape and dimensions fit between the imprinted cavities and the NPs to be detected. The finding that unspecific Van der Waals attractive forces [87] and entropic effects [93] promote the landing of NPs in the cavities of NPIP points towards the attainment of this goal.

It has also been shown that bulk SiO_2_ i-NPIP materials can quantitatively uptake NPs from water [87]. In this example, the uptaken NPs (AgNPs) were of the same nature as those used for imprinting (AgNPs and AuNPs), but the consideration that unspecific attractive forces between cavities and NPs hold in this material suggests its use for the recovery of NPs of multiple different materials from the environment. A perspective in this area is, for sure, the design of bulk devices (e.g., flow channels and filters), in which this kind of material is used for the treatment of surface water for the removal of a large range of nanomaterials, including nanoplastics.

Finally, the size selectivity is seen as a value in the sensing science. However, sensors capable of detecting simultaneously the NPs of a wide range of sizes, as well as bulk materials capable of doing the same in removing NPs from surface water or air, should be a long-term goal of this research area. In this regard, bulk SiO_2_ i-NPIP imprinted simultaneously with a set of NPs of a wide range of different dimensions are currently under investigation in our labs.

## Figures and Tables

**Table 1:** Reviewed NPIP-based sensors for NPs. The table reports the NPs materials and dimensions, the sensing technique, the imprinted polymer, the parameter on which selectivity is based, and the detection range.

Sensed NPs Material and Dimensions ^a^	Sensing Technique ^b^	NPIP Material ^c^	Selectivity ^d^	Detection Range ^d^	Ref.
**AgNPs** 60 nm	QCM	PU, PS, and PVA	n.d.	68–270 ppm	[67]
40 nm	LSV and ITO electrode	PPh	dimensions	n.d.	[70]
10 nm	LSV and ITO electrode	PEI and OA	dimensions	n.d.	[71]
**AuNPs** 15 nm, 33 nm	LSV and ITO electrode	PANI	dimensions	n.d.	[68]
2 nm	LSV and ITO electrode	CA	coating	n.d.	[69]
10 nm, 40 nm	LSV and ITO electrode	PEI, OA	dimensions and coating	n.d.	[71]
8 nm	LSV and ITO electrode	silica gel	dimensions	n.d.	[72]
8.5 nm	LSV and ITO electrode	polymerized p-ADA salts	n.d.	n.d.	[73]
15 nm	LSV and ITO electrode	polymerized CBD	coating	n.d.	[74]
55 nm	LSV and glassy carbon electrode	polymerized plumbagin	dimensions and coating	n.d.	[75]
47 × 12 nm, 34 × 13 nm rods	LSV and ITO electrode	polymerized CBD or ADB	dimensions and charge	n.d.	[76]
**QDs** CdTeSe/ZnS 18 × 7 nm rods	reflectance	PMAA	dimensions, shape, and coating	0.025–25 ppb	[77]
CdSSe/ZnS 5–10 nm	QCM	PMAA/ EGDMA	dimensions and coating	25–2000 ppb	[78]
**SiO_2_NPs** 30 nm	SWV and Au electrode	PPh	n.d.	n.d.	[79]
100 nm	capacitance and Au IDE	SiO_2_	dimensions	n.d.	[80]

^a^ unless otherwise stated, the NP’s shape is spherical; ^b^ QCM = quartz crystal microbalance; LSV = linear sweep voltammetry; SWV = square wave voltammetry; IDE = interdigitated electrode. ^c^ PU = polyurethane; PS = polystyrene; PVA = polyvinyl alcohol; PPh = polyphenol; PEI = polyethylene imine; OA = oleic acid; PANI = polyaniline; CA = cellulose acetate; p-ADA = p-aryldiazonium; CBD = 4-carboxybenzenediazonium; ADB = 4-acetylbenzenediazonium; PMAA = poly(methacrylic acid); EGDMA = ethylene glycol dimethacrylate. ^d^ n.d. = not determined.
